# Cardiac Arrest in a Young Soccer Player With a Pathogenic *SCN5A* Variant

**DOI:** 10.1016/j.jaccas.2026.108906

**Published:** 2026-07-15

**Authors:** Ian R. Sigal, John Power, Amy R. Kontorovich, Daniel Musikantow

**Affiliations:** a Icahn School of Medicine at Mount Sinai, New York, New York, USA; b Mount Sinai Fuster Heart Hospital, New York, New York, USA; c Helmsley Electrophysiology Center, New York, New York, USA

**Keywords:** electrophysiology, exercise, genetics, secondary prevention

## Abstract

**Background:**

The *SCN5A* gene encodes the alpha subunit of the cardiac sodium channel; variants are associated with inherited cardiac diseases including long QT syndrome, sick sinus syndrome, dilated cardiomyopathy, arrhythmogenic cardiomyopathy, and Brugada syndrome.

**Case Summary:**

A 19-year-old male soccer player with a family history of sudden cardiac death collapsed during practice and received an automated external defibrillator shock for polymorphic ventricular tachycardia. Genetic testing identified a pathogenic *SCN5A* c.4222G>A (p.Gly1408Arg) variant previously associated with Brugada syndrome, although no Brugada pattern was identified on electrocardiogram. A subcutaneous implantable cardioverter-defibrillator was placed. After 8 months without arrhythmias and a reassuring exercise stress test, the patient returned to competitive sports through shared decision-making.

**Discussion:**

This case highlights the value of genetic testing after unexplained cardiac arrest and challenges surrounding return-to-sport decisions in inherited arrhythmia syndromes.

**Take-Home Messages:**

Consider genetic testing in cases of unexplained sudden cardiac arrest. Accessible automated external defibrillators are essential for safe athletic environments.

## Visual Summary

**Figure:**
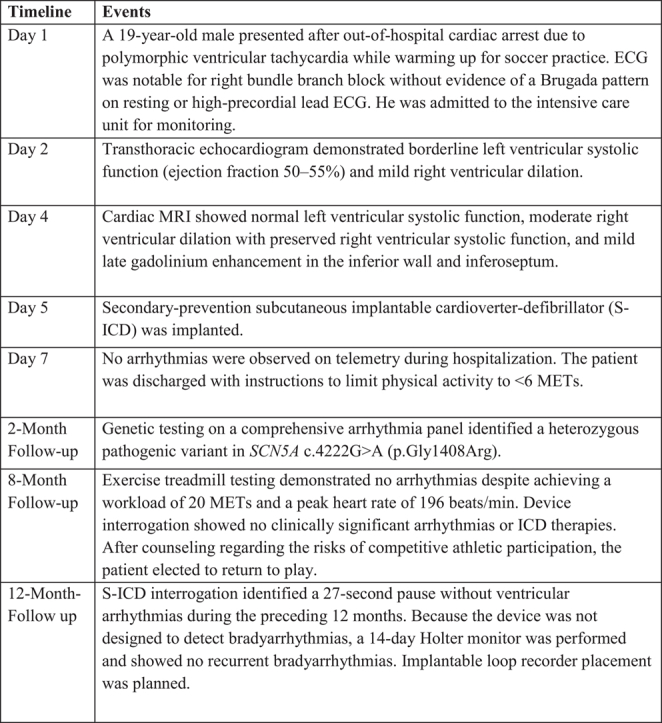

## History of Presentation

**Figure 1:**
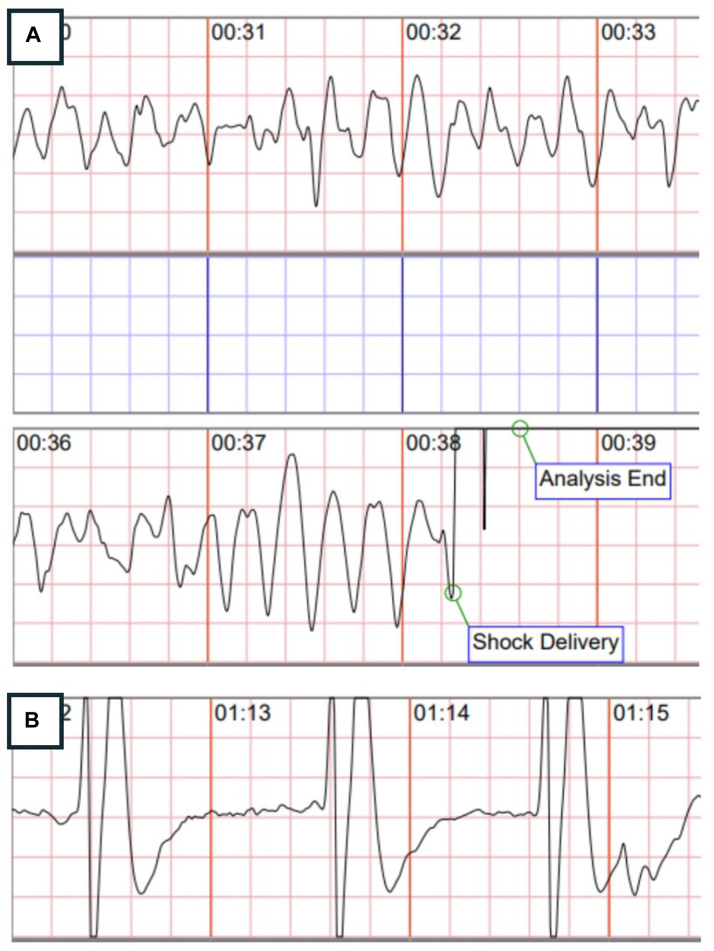
AED Recording (A) AED recording demonstrating polymorphic ventricular tachycardia. (B) After an appropriate shock, the patient returned to sinus rhythm. AED = automated external defibrillator.

A 19-year-old male college soccer player was admitted to the hospital after a witnessed cardiac arrest. While warming up before soccer practice at an ambient temperature of 13 °C, the patient experienced sudden lightheadedness and collapsed. On-site emergency medical services initiated cardiopulmonary resuscitation (CPR). An automated external defibrillator (AED) was brought to the scene and delivered an appropriate shock (Figure 1) for polymorphic ventricular tachycardia, with return of sinus rhythm. Upon hospital arrival, the patient was alert and oriented without neurological deficits; vital signs and physical examination were unremarkable.

**Take-Home Messages**

• Clinicians should consider genetic testing in cases of unexplained sudden cardiac arrest to guide management and enable screening of at-risk relatives.
• Emergency action plans and rapidly accessible automated external defibrillators are essential to maintain a safe athletic environment.

## Past Medical History

The patient reported 2 prior syncopal episodes: one after playing soccer in hot weather 2 years earlier, and another during a febrile illness 6 months before presentation. He had no prior cardiac work-up.

**Figure 2:**
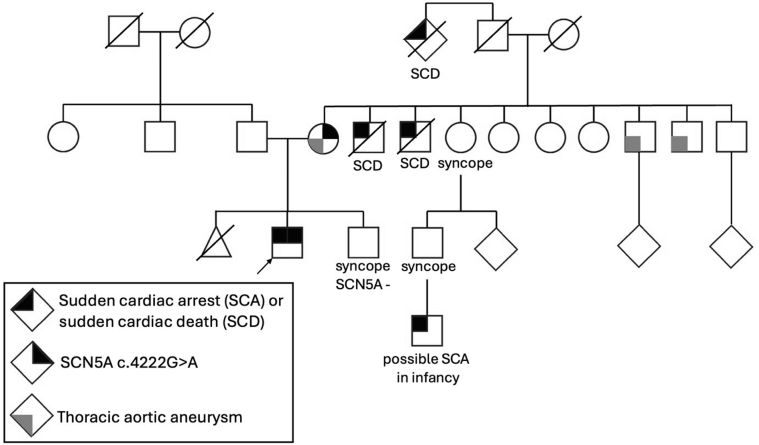
Family Pedigree SCA = sudden cardiac arrest; SCD = sudden cardiac death.

Family history was notable for sudden nocturnal cardiac death in 2 maternal uncles at ages 6 and 18. His brother had previously experienced syncope during viral illness. His mother and multiple maternal uncles had been diagnosed with ascending aortic aneurysms, 1 requiring surgical repair (Figure 2).

## Differential Diagnosis

In a young athlete with exertional syncope and a family history of sudden unexplained death, the differential diagnosis included catecholaminergic polymorphic ventricular tachycardia, long QT syndrome, arrhythmogenic cardiomyopathy (ACM), Brugada syndrome, and myocarditis.

## Investigation

**Figure 3:**
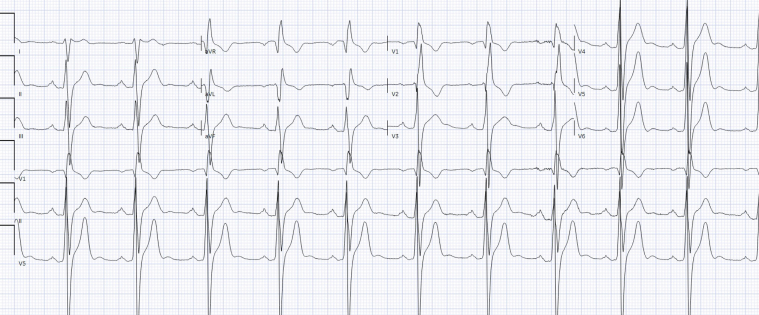
Admission Electrocardiogram Demonstrating Right Bundle Branch Block

**Figure 4:**
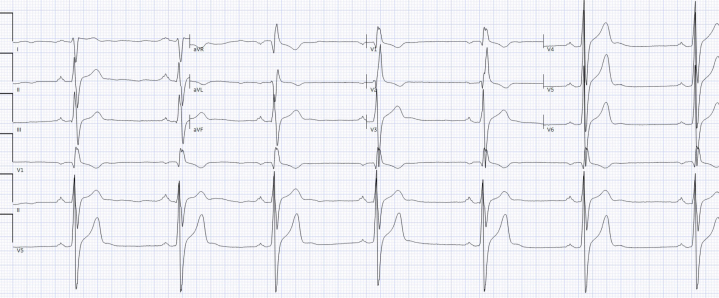
Electrocardiogram With High Lead Placement

The patient's initial electrocardiogram (ECG) demonstrated sinus rhythm with right bundle branch block (Figure 3), and a Brugada pattern was not apparent on a repeat ECG with high lead placement (Figure 4). Admission laboratories were notable for high-sensitivity troponin I of 4.30 ng/L, thyroid stimulating hormone of 6.489 mIU/L with normal T4 of 1.16 ng/dL, and serum potassium of 2.9 mEq/L; results were otherwise unremarkable.

**Figure 5:**
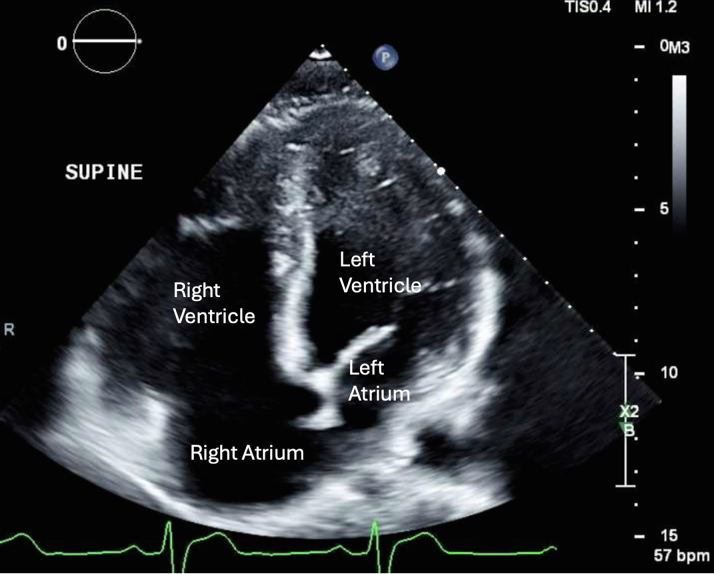
Apical 4-Chamber View Demonstrating Mild Right Ventricular Dilation

Transthoracic echocardiogram performed 1 day after resuscitation demonstrated borderline left ventricular systolic function (ejection fraction: 50%-55%) and mild right ventricular (RV) dilation (Figure 5). Cardiac magnetic resonance imaging performed 4 days after resuscitation was notable for normal left ventricular systolic function and moderate RV dilation (end-diastolic volume: 122 mL/m^2^), with normal RV systolic function and mild late gadolinium enhancement in the inferior wall and inferoseptum. The interpreting radiologist also noted a possible focus of RV dyskinesis but reported that this equivocal finding did not clearly meet the threshold for a regional wall motion abnormality under the 2020 Padua international diagnostic criteria. The patient had no T-wave inversions, epsilon waves, or ventricular extrasystoles that would satisfy additional major or minor Padua criteria for right ventricular ACM.^1^ The patient was monitored on telemetry throughout his hospital course, without arrhythmias.

Genetic testing on a comprehensive arrhythmia panel (100 genes) identified a heterozygous pathogenic variant in *SCN5A* c.4222G>A (p.Gly1408Arg). No additional variants were reported. This rare loss-of-function variant has previously been reported in multiple unrelated individuals with autosomal-dominant Brugada syndrome.^2^ Consistent with the known pleiotropy of *SCN5A*, this variant has also been associated with familial sick sinus syndrome.^3^ In the context of the patient's cardiac arrest and *SCN5A* variant, Brugada syndrome was considered the most likely diagnosis, although unconfirmed in the absence of a Brugada pattern on ECG. Procainamide challenge was deferred during this admission. Single-site cascade testing revealed maternal inheritance of the *SCN5A* variant; the patient's brother tested negative.

## Management

A secondary-prevention subcutaneous implantable cardioverter-defibrillator (S-ICD) was placed before discharge. The patient was advised to restrict physical activity to 6 metabolic equivalents (METs) given his exertion-related cardiac arrest.

## Outcome and Follow-Up

After 8 months, a maximal-effort Bruce protocol stress test demonstrated excellent exercise capacity (peak workload: 20 METs, peak heart rate: 196 beats/min) without ventricular ectopy or inducible arrhythmias.

The patient wished to return to collegiate soccer. He was counseled regarding the increased risk of appropriate and inappropriate ICD shocks, uncertain long-term data on athletic participation with pathogenic *SCN5A* variants, and potential device-related trauma. Through shared decision-making, he elected to resume competitive play. Before return, AED access at training venues and CPR training for coaching staff were confirmed. Given suspicion for Brugada syndrome, risk mitigation strategies included avoidance of arrhythmogenic medications, dehydration, heat exposure, and electrolyte depletion. His S-ICD was programmed with a conditional ventricular tachycardia detection zone of 220 to 240 beats/min and a shock zone for rates >240 beats/min to minimize inappropriate therapy while maintaining sensitivity for malignant ventricular arrhythmias.

After return to sport, S-ICD interrogation revealed a possible 27-second pause detected incidentally through the SMART Pass sensing filter algorithm. No recurrent pauses were noted on 14-day Holter monitor. Because this was an isolated event and his S-ICD cannot reliably detect bradyarrhythmias, loop recorder implantation is planned for ongoing monitoring. The patient has experienced no ICD therapies or recurrent symptoms since his initial event.

## Discussion

The *SCN5A* gene encodes the alpha subunit of the cardiac sodium channel; *SCN5A* variants are associated with cardiac pathologies including Brugada syndrome, familial sick sinus syndrome, congenital long QT syndrome, dilated cardiomyopathy, and ACM. The c.4222G>A variant has been reported in patients with Brugada syndrome^2^ and familial sick sinus syndrome.^3^

Brugada syndrome is a Mendelian inherited sodium-channel disorder with an estimated prevalence of 1 in 5,000;^4^ despite the strong association with *SCN5A*, most patients with clinically diagnosed Brugada syndrome lack an identifiable genetic variant.^4^ Brugada syndrome is diagnosed by a characteristic type 1 Brugada pattern on ECG; when the initial ECG is negative, the Brugada pattern may be unmasked through provocative testing such as procainamide challenge. Although our patient did not demonstrate a Brugada pattern on resting ECG or during exercise stress testing, the circumstances surrounding his cardiac arrest, family history, and *SCN5A* genotype were suggestive, though not diagnostic, of Brugada syndrome. ACM was also considered given the equivocal magnetic resonance imaging findings; however, the patient met no major or minor ECG criteria for right ventricular ACM under the 2020 Padua criteria. Moreover, to our knowledge, the *SCN5A* c.4222G>A variant has not previously been reported in association with ACM. Although ACM cannot fully be excluded, the overall picture was more consistent with suspected Brugada syndrome. Procainamide challenge was deferred given our strong clinical suspicion for Brugada syndrome as the unifying diagnosis, and the decision was made to manage the patient empirically for this condition. Given the known pleiotropy of *SCN5A*, he will continue ongoing monitoring for conduction disease and cardiomyopathy.

Lethal arrhythmias in patients with Brugada syndrome most commonly occur during rest or sleep, in the context of fever, electrolyte disturbances, or sodium-channel-blocking medications.^4^ Sudden death or syncope during exercise is uncommon in Brugada syndrome, but it has been described in prior case reports and has been associated with ECG abnormalities during exercise stress testing such as J point elevation,^5^ ventricular tachycardia,^6^ or uncovering of a type 1 Brugada pattern.^7^ In contrast, our patient—whose Brugada syndrome diagnosis remains unconfirmed—had no arrhythmias or ECG changes during stress testing, and his concomitant hypokalemia could have contributed to his arrest independently of exertion.

Familial sick sinus syndrome is also associated with the c.4222G>A variant and may explain his possible sinus pause. Although less likely, the patient's polymorphic ventricular tachycardia arrest may have been provoked by a prolonged R-R interval due to sinus bradycardia with subsequent R-on-T phenomenon.

Identification of this *SCN5A* variant provided a plausible etiology of the patient's arrest, informed the discussions regarding future sports participation, guided potential therapeutics, and enabled screening of at-risk relatives—including his brother, a collegiate athlete with unexplained syncope. Genetic testing and counseling receive Class I recommendations in the 2022 European Society of Cardiology and 2017 American Heart Association/American College of Cardiology guidelines for suspected inherited arrhythmia syndromes with risk of sudden cardiac death.^8^^,^^9^

Rapid AED access and immediate CPR were essential to this patient's survival and neurologic recovery, reinforcing the necessity of well-executed emergency action plans in athletic environments.

Sports participation in patients with symptomatic Brugada syndrome remains controversial. Although malignant arrhythmias in Brugada syndrome are not adrenergically mediated, athletes may experience increased parasympathetic tone during recovery or exercise-induced hyperthermia, both potential arrhythmia triggers.^10^ Historically, these concerns led to broad disqualification from competitive sports. These risks remain hypothetical, and data supporting sports restriction are lacking.^10^^,^^11^ Sports restriction is also not warranted for genotype-positive, phenotype-negative individuals who harbor pathogenic *SCN5A* variants but do not have features of any associated disease (ie, asymptomatic relatives uncovered through cascade screening).

Accordingly, recent American Heart Association/American College of Cardiology and European Society of Cardiology guidelines support competitive sports participation for selected athletes with symptomatic Brugada syndrome following ICD implantation and shared decision-making, while emphasizing avoidance of arrhythmic triggers such as fever, heat exposure, and dehydration.^11^^,^^12^ In contrast, the Italian Society of Sports Cardiology continues to advise against competitive athletics for patients with symptomatic Brugada syndrome, even after ICD implantation.^13^ In the absence of evidence supporting exercise restrictions, return-to-sport decisions require individualized assessment. In this case, the patient's event during low-intensity exertion, stable follow-up, reassuring exercise testing, and personal goals supported a reasonable shared decision to resume participation in college soccer despite his suspected Brugada syndrome diagnosis.

## Conclusions

Genetic evaluation is essential in young athletes with unexplained cardiac arrest, guiding management and enabling family screening. Return-to-sport decisions for patients with pathogenic *SCN5A* variants require individualized assessment and shared decision-making given the limited evidence base. This case also underscores the impact of rapid AED deployment and emergency action protocols in athletic environments.

## Funding Support and Author Disclosures

Dr Kontorovich has received research support from Pfizer Inc. All other authors have reported that they have no relationships relevant to the contents of this paper to disclose.

**Visual Summary:** Timeline of Events

Time	Events
Day 1	A 19-year-old male athlete presented after out-of-hospital cardiac arrest due to polymorphic ventricular tachycardia while warming up for soccer practice. ECG was notable for right bundle branch block without evidence of a Brugada pattern on resting or high-precordial lead ECG. He was admitted to the intensive care unit for monitoring.
Day 2	Transthoracic echocardiogram demonstrated borderline left ventricular systolic function (ejection fraction: 50%-55%) and mild right ventricular dilation.
Day 4	Cardiac MRI showed normal left ventricular systolic function, moderate right ventricular dilation with preserved right ventricular systolic function, and mild late gadolinium enhancement in the inferior wall and inferoseptum.
Day 5	Secondary-prevention S-ICD was implanted.
Day 7	No arrhythmias were observed on telemetry during hospitalization. The patient was discharged with instructions to limit physical activity to <6 METs.
2-mo follow-up	Genetic testing on a comprehensive arrhythmia panel identified a heterozygous pathogenic variant in *SCN5A* c.4222G>A (p.Gly1408Arg).
8-mo follow-up	Exercise treadmill testing demonstrated no arrhythmias despite achieving a workload of 20 METs and a peak heart rate of 196 beats/min. Device interrogation showed no clinically significant arrhythmias or ICD therapies. After counseling regarding the risks of competitive athletic participation, the patient elected to return to play.
12-mo follow up	S-ICD interrogation identified a 27-second pause without ventricular arrhythmias during the preceding 12 moths. Because the device was not designed to detect bradyarrhythmias, a 14-day Holter monitor was performed and showed no recurrent bradyarrhythmias. Implantable loop recorder placement was planned.

ECG = electrocardiogram; MRI = magnetic resonance imaging; S-ICD = subcutaneous implantable cardioverter-defibrillator.
